# Derivation method of the dielectric function of amorphous materials using angle-resolved electron energy loss spectroscopy for exciton size evaluation

**DOI:** 10.1093/jmicro/dfae056

**Published:** 2025-02-04

**Authors:** Tomoya Saito, Yohei K Sato, Masami Terauchi

**Affiliations:** Institute of Multidisciplinary Research for Advanced Materials, Tohoku University, 2-1-1, Katahira, Aobaku, Sendai 980-8577, Japan; Institute of Multidisciplinary Research for Advanced Materials, Tohoku University, 2-1-1, Katahira, Aobaku, Sendai 980-8577, Japan; Institute of Multidisciplinary Research for Advanced Materials, Tohoku University, 2-1-1, Katahira, Aobaku, Sendai 980-8577, Japan

**Keywords:** angle-resolved electron energy loss spectroscopy, amorphous materials, amorphous SiO_2_, dielectric function, exciton size, photocatalysis

## Abstract

Accurately deriving the momentum transfer dependence of the dielectric function *ε*(***q***, *ω*) using angle-resolved electron energy loss spectroscopy (AR-EELS) is necessary for evaluating the average electron–hole distance, i.e. the exciton size, in materials. Achieving accurate exciton size evaluations will promote the comprehension of optical functionality in materials such as photocatalysts. However, for amorphous materials, it is difficult to accurately derive *ε*(***q***, *ω*) because the elastic scattering intensity originating from the amorphous structure and the inelastic scattering intensity associated with elastic scattering overlap in the EELS spectrum. In this study, a method to remove these overlapping intensities from the EELS spectrum is proposed to accurately derive *ε*(***q***, *ω*) of an amorphous material. Amorphous SiO_2_ (am-SiO_2_) was subjected to AR-EELS measurements, and *ε*(***q***, *ω*) of am-SiO_2_ was derived after removing the intensity due to the amorphous structure using the proposed method. Thereafter, the exciton absorption intensity and the exciton size were evaluated. Applying the proposed method, the exciton absorption intensity was considerably suppressed in the *q*-region after 1.0 Å^−1^, where the elastic and inelastic scattering intensities originating from the amorphous structure are dominant. The exciton size evaluated was 2 nm ($ \pm $ 1 nm), consistent with the theoretically predicted size of ∼1 nm. Therefore, the proposed method is effective for deriving accurate *ε*(***q***, *ω*), facilitating exciton size evaluation for amorphous materials using AR-EELS.

## Teaser

A method for deriving the momentum transfer-dependent dielectric function of amorphous materials by angle-resolved EELS is proposed, and exciton sizes are evaluated. The proposed method will enable the evaluation of exciton size of amorphous materials, and is expected to be useful for elucidating the mechanism of optical functionality

## Introduction

In optical functional materials such as solar energy materials [1–3], photocatalytic materials [4–6], and photo devices [7–9], optically excited electron–hole (e–h) pairs, i.e. excitons, play an important role. Various studies have been conducted to characterize excitons to understand and consequently improve the functionality of optical functional materials. Accordingly, the diffusion length and lifetime of excitons have been established as the key factors governing the functionality of solar cells and photocatalytic materials [10–14]. These factors determine the ease with which excitons can reach the heterojunction where e–h pair separation occurs in solar cell materials. In photocatalytic materials, these factors affect the ease with which excitons can reach the crystal surface where the photocatalytic reaction occurs.

In a previous study, the exciton size was demonstrated to be a key factor affecting the performance of photocatalytic materials [15]. In that study, the exciton size of single-crystalline titanium dioxide (anatase) was experimentally evaluated using angle-resolved electron energy loss spectroscopy (AR-EELS) based on transmission electron microscopy (TEM). The values of the exciton size were consistent with the values predicted by a combination of optical absorption measurements and *ab initio* calculations [16]. Additionally, it has also been mentioned that the orientation of the photocatalytically active crystal surface is affected by the anisotropy of the exciton size. Thus, exciton size evaluation has attracted considerable research interest. Unfortunately, few reports on exciton size evaluation for optical functional materials in practical use exist. Therefore, evaluating the exciton size in various optical functional materials and exploring the correlation between the exciton size and their functionality are imperative. Although the exciton size is traditionally estimated using photoluminescence [17,18] and magnetic absorption measurements [19,20], these methods offer macroscopic evaluations only for bulk materials. Conversely, AR-EELS based on TEM can evaluate individual particles. Considering that most optical functional materials in practical use are submicron-scale particles, AR-EELS based on TEM is an indispensable spectroscopic technique.

Crystalline materials as well as amorphous materials have attracted attention due to their potential for use as optical functional materials. For example, amorphous TiO_2_ is expected to offer improved photocatalytic activity over crystalline TiO_2_ [21,22]. This is believed to be due to the increased specific surface area resulting from amorphization, which allows molecules to be adsorbed more easily. Amorphous Si has been applied in solar cells [23,24], and it has a higher optical absorption efficiency than crystalline Si, allowing the production of low-cost and high-efficiency cells [25]. However, the exciton size in amorphous materials has not been examined (including by AR-EELS).

To estimate the exciton size by AR-EELS, it is necessary to derive the absorption intensity (imaginary part of the dielectric function) depending on the momentum transfer *q* for the material. It can be derived from the bulk loss function Im[−1/*ε*] by Kramers–Kronig (KK) analysis. However, to derive a more accurate Im[−1/*ε*], it is necessary to obtain a single-scattering distribution by removing intensities owing to the multiple scattering of bulk excitations, surface plasmon polaritons and Cherenkov radiation [26,27]. The multiple scattering intensities can be removed using the Fourier-log deconvolution method [28]. Intensities owing to surface plasmon polaritons and Cherenkov radiation can be avoided in the analysis with *q*  ≠  0, because these loss intensities decay more rapidly with increasing *q* [29]. Furthermore, two other types of overlapping intensities exist for amorphous materials. One is the elastically scattered intensity broadly distributed in the *q*-space as a broad-ring intensity in the electron diffraction pattern. The second overlapping intensity originates from the inelastic scattering experienced by the elastically scattered electrons inside the material. For quantitative analysis, these two additional overlapping intensities must also be removed. However, thus far, no analytical procedure for removing these intensities has been proposed.

In this study, a method for removing these two additional intensities originating from the amorphous structure was proposed and applied to amorphous SiO_2_ (am-SiO_2_) for exciton size evaluation. Furthermore, the proposed method was validated, focusing on its performance in the experimental derivation of the dielectric function and the exciton size evaluation.

## Experimental

Cover glasses, i.e. borosilicate glass (Matsunami Glass Ind., Ltd.), were employed as the am-SiO_2_ specimen. The specimens for the TEM observation and AR-EELS measurement were prepared by crushing the cover glass and placing fine segments on the microgrid for TEM observations.

AR-EELS measurements were performed using JEM-2010FEF (JEOL) equipped with a monochromator at an acceleration voltage of 100 kV [30]. A *q*-selection slit (1.5 × 50 µm^2^) was inserted in the diffraction plane to select the momentum transfer of the inelastically scattered electrons. The longer edge of the *q*-selection slit was perpendicularly aligned to the energy dispersion of the analyzer used. Therefore, the inelastically scattered electrons passing through the *q*-selection slit formed a two-dimensional intensity distribution representing momentum transfer (*q*) on the horizontal axis and energy loss (*E*) on the vertical axis, i.e. the *E*–*q* map [15]. The energy and *q* (longitudinal direction of the slit) resolutions are approximately 0.3 eV and 0.1 Å^−1^, respectively. The *q*-resolution in the short-side-edge direction of the slit is ∼0.1 Å^−1^, which is estimated as the slit width in reciprocal space. The *q*-resolution in the direction parallel to the incident electron beam on the specimen is expressed as $\Delta {q_z} = {q_x} \cdot \tan \alpha $ using the convergence angle of the incident beam, *α* = 14 mrad, and the *q* value in the longer slit direction, *q_x_* [31]. *q_x_* was adopted as the maximum *q* value in the *E*–*q* map, 3 Å^−1^, and $\Delta {q_z}$ ∼ 0.04 Å^−1^. The diameter of the electron probe for the specimen was 120 nm. Imaging plates were employed as a detector. The exposure time for one *E*–*q* map was 6 min, with 10 exposures added to improve the signal-to-noise ratio.

## Results and discussion

### TEM observation

**Fig. 1.**
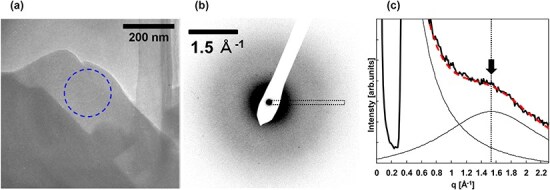
(a) TEM image, (b) diffraction pattern and (c) the black-dotted-line profile for (b). The thick solid line in (c) is the line profile intensity. The thin solid line is the Gaussian fitted to the transmitted and halo intensity distribution. The dashed line is the linear sum of the Gaussians.

Figure 1a and b shows a TEM image and an electron diffraction pattern of the am-SiO_2_ specimen, respectively, for the AR-EELS measurements. The electron diffraction pattern was obtained from the 180-nm-diameter area (circled by the dashed line) in Fig. 1a. The specimen thickness in the measurement area was estimated to be 50 nm using the log-ratio method (see Supplementary Data 1). An elastically scattered halo ring intensity caused by the amorphous structure can be observed in Fig. 1b.

Figure 1c shows the intensity profile of the area indicated by the dotted rectangle in Fig. 1b. The dashed line indicates the profile fitted using two Gaussians of the transmitted beam and amorphous halo intensity represented by thin lines. The fitted result shows that the peak of the amorphous halo intensity (arrow in Fig. 1c) is 1.53 Å^−1^. This result is consistent with the X-ray diffraction result on am-SiO_2_, in which the first sharp diffraction peak appears at 1.52 Å^−1^ [32]. The value in the reciprocal space corresponding to 4.1 Å in real space mainly reflects the distances to the second-nearest-neighbor atoms, such as Si–Si_2nd_, O–O_2nd_, and Si–O_2nd_, which contribute to the network structure of interconnected SiO_4_ tetrahedral in am-SiO_2_ [33].

### AR-EELS measurement

**Fig. 2.**
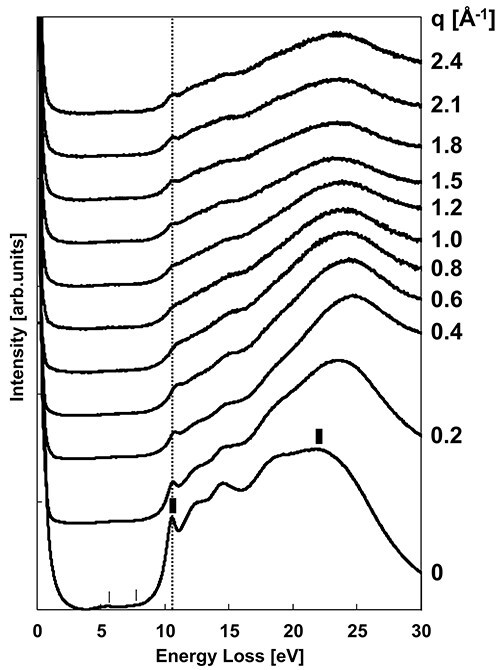
*q*-dependence of the EELS spectra.

Figure 2 shows the *q*-dependence of the EELS spectra. These spectra were acquired with step widths of Δ*q* = 0.2 Å^−1^ for the range of 0 Å^−1^ ≤ *q* ≤ 1.0 Å^−1^ and Δ*q* = 0.3 Å^−1^ for 1.2 Å^−1^ ≤ *q* ≤ 2.4 Å^−1^, where Δ*q* was varied for the large *q*-region to maintain a reasonable signal-to-noise ratio.

The peak of *q* = 0 Å^−1^ at 10.5 eV, indicated by a thick line, is attributable to a free exciton [34]. The corresponding peak or shoulder structure was observed at almost the same energy at other *q* values, suggesting that the structure corresponds to free excitons. This structure became indistinct as *q* increased to 1.0 Å^−1^ and was gradually distinct as *q* exceeded 1.0 Å^−1^. This is because the inelastic scattering intensity distribution after *q* = 1.0 Å^−1^ is dominated by the superposition of the inelastic scattering intensity associated with the elastic scattering originating from the amorphous structure. The diffraction intensity in Fig. 1c shows that the amorphous halo intensity becomes dominant just after *q* = 1.0 Å^−1^. The faint structures at 6 and 8 eV, indicated by thin lines in the *q* = 0 spectrum, correspond to the absorption structure due to oxygen deficiency in am-SiO_2_ [35]. At even lower energies, no major structure is observed, except for the zero-loss tail, and the contribution from Cherenkov radiation should be negligible. Cherenkov radiation occurs at acceleration voltages of 100 kV for refractive index *n* > 1.8 [28], while am-SiO_2_ has *n* ∼ 1.5 [36–38] and no radiation occurs. The broad peak at 22 eV in the *q* = 0 spectrum could correspond to a volume plasmon [34]. The peak energy is smaller than that for *q* > 0.4 Å^−1^ of ∼24 eV and comparable to the energy of 22 eV observed in reflection EELS [35]. This suggests that the effect of surface loss cannot be neglected in the *q* = 0 spectrum in this study.

The volume plasma frequency, ${\omega _{\mathrm{p}}}$, is expressed as ${\omega _{\mathrm{p}}} = \sqrt {\frac{{4{{\pi }}{e^2}N}}{m}} $ using the valence electron density *N*, elementary charge *e* and electron mass *m*. There are four valence electrons in the constituent atoms for Si (3s^2^, 3p^2^) atoms and six for O (2s^2^, 2p^4^) atoms. Here, since the atomic density (*ρ*) of am-SiO_2_ is frequently found in the range of 2.0–3.0 (${\mathrm{g\;c}}{{\mathrm{m}}^{ - 3}}$) [36–38], *ρ* = 2.5 (${\mathrm{g\;c}}{{\mathrm{m}}^{ - 3}}$) was adopted. Assuming that the composition of the am-SiO_2_ specimen is Si:O = 1:2 homogeneously and since a single molecule of SiO_2_ weighs $1.0{\ } \times {10^{ - 22}}$ g, the valence electron density *N* was calculated to be 4.0 $ \times {10^{23}}{\mathrm{\;c}}{{\mathrm{m}}^{ - 3}}$. Thus, the volume plasma frequency was calculated as ${\omega _{\mathrm{p}}} = 5.7 \times {10^{15}}$ rad s^−1^. This result corresponds to the plasmon energy of 23.4 eV, which is approximately consistent with the experimentally observed value.

### Removal of *I*_amo_(*q*, *E*) owing to the amorphous structure

The experimental *q*-dependent EELS spectral intensity for amorphous materials *I*_EELS_ (***q***, *E*) is expressed as follows:


$${I_{{\mathrm{EELS}}}}\left( {q,{\mathrm{\;}}E} \right) = {\mathrm{\;}}{I_{{\mathrm{trans}}}} + {I_{{\mathrm{inela}}}}\left( {q,{\mathrm{\;}}E} \right) + {I_{{\mathrm{amo}}}}\left( {q,{\mathrm{\;}}E} \right),$$

$${I_{{\mathrm{amo}}}}\left( {q,{\mathrm{\;}}E} \right) = {I_{{\mathrm{amo}}\_{\mathrm{ela}}}}\left( {q,{\mathrm{\;}}0} \right) + {I_{{\mathrm{amo}}\_{\mathrm{ela}} + {\mathrm{inela}}}}\left( {q,{\mathrm{\;}}E} \right),$$

where *I*_trans_ is the transmitted beam intensity. *I*_inela_(***q***, *E*) is the inelastic scattering intensity without elastic scattering. *I*_amo_(***q***, *E*) is the overlapping intensity and is expressed as the sum of the elastic scattering intensity due to the amorphous structure *I*_amo_ela_(***q***, 0) and the inelastic scattering intensity associated with the elastic scattering *I*_amo_ela+inela_(***q***, *E*).

As mentioned in the Introduction section, to derive accurate dielectric functions, it is necessary to remove the overlapping intensity, *I*_amo_(***q***, *E*), from the *I*_EELS_(***q***, *E*). In the following analysis, *I*_trans_ is also removed and the final analysis is applied to *I*_inela_(***q***, *E*). The ratio of *I*_amo_ela+inela_(***q***, *E*) to I_amo_ela_(***q***, 0) is assumed to correspond to the ratio of the inelastic scattering intensity (*I*_inela_(0, *E*)) to the transmitted beam intensity (*I*_trans_),


$$\frac{{{I_{{\mathrm{amo}}\_{\mathrm{ela}} + {\mathrm{inela}}}}\left( {q,{\mathrm{\;}}E} \right)}}{{{I_{{\mathrm{amo}}\_{\mathrm{ela}}}}\left( {q,{\mathrm{\;}}0} \right)}} = {\mathrm{\;}}\frac{{{I_{{\mathrm{inela}}}}\left( {0,{\mathrm{\;}}E} \right)}}{{{I_{{\mathrm{trans}}}}}}.$$

*I*_inela_(0, E) and *I*_trans_ are, respectively, defined as follows:

${I_{{\mathrm{inela}}}}\left( {0,{\mathrm{\;}}E} \right) = \smallint \limits_{{q_E}}^{ - {q_E}} {I_{{\mathrm{inela}}}}\left( {q,{\mathrm{\;}}E} \right){\mathrm{d}}q$ and ${I_{{\mathrm{trans}}}} = \smallint \limits_{{q_E}}^{ - {q_E}} \smallint \limits_{{{\delta }}E/2}^{ - {{\delta }}E/2} {I_{{\mathrm{EELS}}}}\left( {q,{\mathrm{\;}}E} \right){\mathrm{d}}q{\mathrm{d}}E,$,

where *q*_E_ is *k*_0_Δ*E*/2*E*_0_, *k*_0_ is the wavenumber of the incident electron beam, Δ*E* is an energy loss value and *E*_0_ is the acceleration voltage. Since the volume plasmon loss dominates in the low-loss energy regions lower than 50 eV, 22 eV was chosen as the Δ*E* of the volume loss of am-SiO_2_, as shown in Fig. 2. The *k*_0_ value at *E*_0_ = 100 kV was calculated as 2π/*λ* = 169.7 Å^−1^. Next, the *q*_E_ value was calculated to be ∼0.02 Å^−1^, which is one order smaller than the *q*-step values of Δ*q* = 0.2 and 0.3 Å^−1^ in the EELS spectra. Thus, the integral range of −*q*_E_ to *q*_E_ above can be replaced by the *q*-step values of Δ*q*. Integration of *I*_trans_ in the energy direction was performed with an energy resolution of δ*E* = 0.3 eV.

**Fig. 3.**
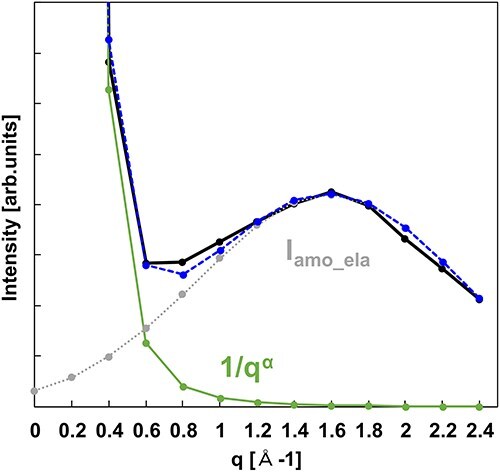
The plot of the experimental zero-loss intensity in *q*-space, black line. Thick- and dotted lines are the fitted zero-loss intensity of 1/*q^α^* (*α* ∼ 4) and Gaussian *I*_amo_ela_, respectively. The dashed line is the sum of the 1/*q^α^* and *I*_amo_ela_ Gaussian.

*I*_amo_ela_(***q***, 0) can be estimated by the following process. Figure 3 shows the plot of the experimental zero-loss intensity in *q*-space. The intensity bulge centered at *q* = 1.6 Å^−1^ is attributed to *I*_amo_ela_(***q***, 0) and corresponds to the broad peak shown in Fig. 1c. The fitting was done assuming that *I*_amo_ela_(***q***, 0) is Gaussian (dotted line) and that the transmitted beam intensity broadening without the elastic scattering intensity (*I*_trans_) is 1/*q^α^* (thick line).

### KK analysis

The surface loss intensity is proportional to 1/*q*^3^, which shows more rapidly decay than that for the bulk loss intensity with 1/*q*^2^ [29]. In this study, the following analysis was performed only for EELS spectra with *q* ≧ 0.4 Å^−1^ to avoid the intensity of surface effects.

After removing *I*_amo_, KK analysis was performed using normal procedures, where the zero-loss peak tail was subtracted by Lorentzian fitting, and the multiple scattering intensities of inter-band transition and plasmon were subtracted using the Fourier-log deconvolution method. The single-scattering distributions were normalized by the sum rule to derive the loss function according to the following equation [28]. As noted in Fig. 2, ${\omega _{\mathrm{p}}}$ is the volume plasma frequency, and the calculated value was employed.


$$ \smallint \limits_0^\infty \omega {\mathrm{Im}}\left[ { - \frac{1}{{\varepsilon (q,\omega )}}} \right]{\mathrm{d}}\omega = \frac{\pi }{2}\omega _p^2.$$

**Fig. 4.**
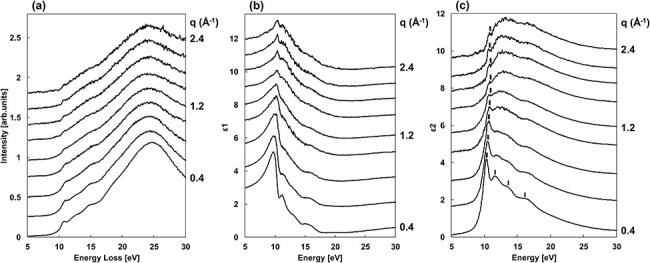
*q*-dependence of (a) the loss functions, (b) the real part of the dielectric functions ${\varepsilon _1}$ and (c) the imaginary part of the dielectric functions ${\varepsilon _2}$.

Figure 4a shows the derived *q*-dependence (*q* ≧ 0.4 Å^−1^) of the loss functions, which are similar in profile to the EELS spectra in Fig. 2, except in the energy region affected by the zero-loss intensity. Figure 4b and c shows the *q*-dependence of the real (${\varepsilon _1}$) and imaginary (${\varepsilon _2}$) part of the dielectric functions derived from the loss function in Fig. 4a using the KK analysis.

**Fig. 5.**
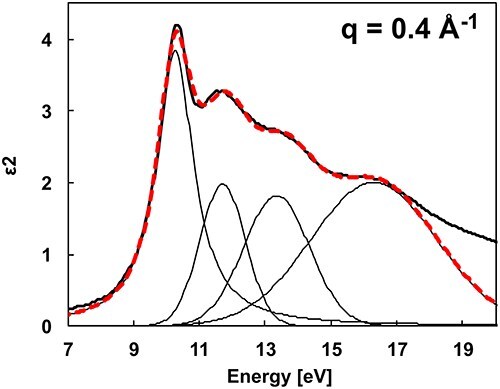
Peak fitting of ${\varepsilon _2}$ using a Lorentzian and three Gaussians.

In Fig. 4b, the peak structures at 10–11 eV, indicated by thick lines, are the absorption peaks of the free exciton in am-SiO_2_ [34]. Focusing on *q* = 0.4 Å^−1^, other absorption structures indicated by thin lines can be observed around 11.6, 13.7, and 16.6 eV. Absorption structures of am-SiO_2_ can be at the same energies [39,40]. Studies have shown that the free exciton absorption peaks in am-SiO_2_ are well reproduced by Lorentzian rather than Gaussian fittings [35]. Thus, each ${\varepsilon _2}$ was fitted in the energy range of 8–18 eV with one Lorentzian for the exciton absorption peak and three Gaussians for the electronic transitions. Figure 5 shows the fitted result for *q* = 0.4 Å^−1^, showing the four fitted peaks and those sums (dashed line). The exciton absorption intensity at each *q* value was estimated by fitting a Lorentzian, expressed as $\frac{{f\omega \gamma }}{{{{\left( {{\omega ^2} - \omega _i^2} \right)}^2} + {{\left( {\omega \gamma } \right)}^2}}}$, where the parameter *f* denotes the oscillator strength of the exciton absorption, *ω_i_* is the exciton absorption energy and *γ* is the half-width. The Gaussian for fitting the electronic transition structure was $A{e^{ - \frac{1}{2}{{\left( {\frac{{{{{\omega }}_{\mathrm{j}}} - {{\omega }}}}{{{\sigma }}}} \right)}^2}}}$. *A* is the fitting parameter for the absorption intensity, *ω_j_* is the absorption energy and *σ* is the standard deviation.

### Exciton size evaluation

**Fig. 6.**
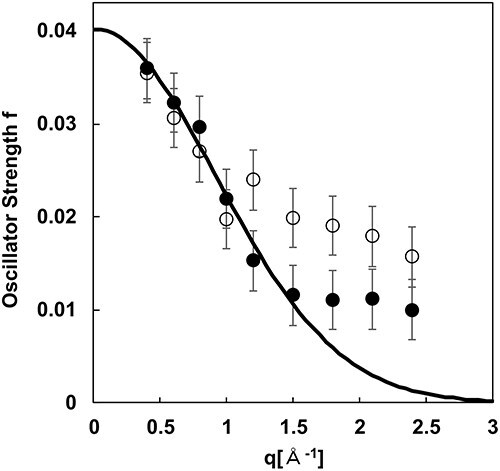
Exciton absorption intensity distribution. The black and white dots show the absorption intensity with and without *I*_amo_ removal, respectively. The solid curve indicates the Gaussian fit to the black dots.

Figure 6 shows the *q*-dependence of the exciton absorption intensities evaluated with (black dots) and without (white dots) the removal of *I*_amo_. For the white dots, the intensity decreases with increasing *q* up to 1.0 Å^−1^; however, the intensity increases for *q* > 1.0 Å^−1^, with a maximum at *q* = 1.8 Å^−1^. Conversely, for the black dots, the intensity for *q* > 1.0 Å^−1^ is considerably suppressed. In Fig. 1c, the elastic and inelastic scattering intensities originating from the amorphous structure are dominant for *q* > 1.0 Å^−1^. This suggests the validity of the change in the exciton absorption intensity induced by the application of the proposed method to remove *I*_amo_.

Focusing on the *q*-dependence of the black dots, the exciton absorption intensity decreases with increasing *q*. Assuming that the maximum exciton absorption intensity is observed at *q* = 0 Å^−1^, this *q*-dependence corresponds to the feature of dipole transition [41]. The upper valence band and the lower condition band in am-SiO_2_ are dominated by the 2p orbital of the O atom and the 3d orbital of the Si atom, respectively, allowing the dipole transitions [38]. Therefore, in this analysis, the exciton size was evaluated by assuming dipole transition in the exciton absorption of am-SiO_2_.

A Gaussian fit (solid line in Fig. 6) was performed on the black plots with the maximum at *q* = 0 Å^−1^. The Fourier-transformed (FT) distribution of the fitted Gaussian should correspond to the exciton distribution in real space. The exciton size was evaluated from the full width at half-maximum of the distribution after FT, denoted as $2\sqrt {2\ln 2} \frac{{2{{\pi }}}}{\sigma }$, using the standard deviation (*σ*) of the fitted Gaussian. In the present analysis, *σ* = 0.81 was derived and the exciton size was estimated to be 2 nm (±1 nm). Here, the free exciton size of am-SiO_2_ in the hydrogen model was theoretically predicted to be ∼1 nm [42], and the theoretical and experimental values are consistent.

The equation, $\frac{{{I_{{\mathrm{amo\_ela}} + {\mathrm{inela}}}}\left( {q,{\mathrm{\;}}E} \right)}}{{{I_{{\mathrm{amo\_ela}}}}\left( {q,{\mathrm{\;}}0} \right)}}{\mathrm{\;}}$ = ${\mathrm{\;}}\frac{{{I_{{\mathrm{inela}}}}\left( {0,{\mathrm{\;}}E} \right)}}{{{I_{{\mathrm{trans}}}}}}$, assumes that the inelastically scattered electrons in ${I_{{\mathrm{amo\_ela}} + {\mathrm{inela}}}}\left( {q,{\mathrm{\;}}E} \right)$ experience the same specimen thickness as for ${I_{{\mathrm{inela}}}}\left( {0,{\mathrm{\;}}E} \right)$. However, the effective specimen thickness for inelastic scattering following elastic scattering is smaller than the original specimen thickness. Assuming that scattering intensities follow a Poisson distribution, intensity of ${I_{{\mathrm{amo\_ela}} + {\mathrm{inela}}}}\left( {q,{\mathrm{\;}}E} \right)$ is proportional to *t'*/*λ*_inela_ exp(−*t'*/*λ*_inela_), where the inelastic mean free path is λ_inela_ = 105 nm (see Supplementary Data 2). Therefore, for the smaller specimen thickness for *I*_amo_ela+inela_(***q***, *E*) compared with that for *I*_inela_(0,*E*), the actual relationship becomes $\frac{{{I_{{\mathrm{amo\_ela}} + {\mathrm{inela}}}}\left( {q,{\mathrm{\;}}E} \right)}}{{{I_{{\mathrm{amo\_ela}}}}\left( {q,{\mathrm{\;}}0} \right)}} < \frac{{{I_{{\mathrm{inela}}}}\left( {0,{\mathrm{\;}}E} \right)}}{{{I_{{\mathrm{trans}}}}}}$ (see Supplementary Data 3). Consequently, the intensity subtracted from the EELS spectrum will also be smaller, potentially leaving a bump intensity ∼1.5–2.5 Å^−1^ in Fig. 6, which may represent the intrinsic absorption intensity of am-SiO_2_. Consequently, the intensity subtracted from the EELS spectrum will also be smaller, potentially leaving a bump intensity ∼1.5–2.5 Å^−1^ in Fig. 6, which may represent the intrinsic absorption intensity of am-SiO_2_.

The possible origins of bump intensity for *q* > 1.5 Å^−1^ are multipole transitions of higher order than dipole (e.g. quadrupole transition). The absorption distribution of multipole transitions in *q*-space were calculated in the previous study [41], in which the transition matrix element $f{\mathrm{|exp}}\left( {iqr} \right){\mathrm{|}}i$ were expanded by Laurent series to *n* order terms reflecting dipole (*n* = 1), quadrupole (*n* = 2), and higher terms (*n* = 3, 4…), as a function of non-dimensional parameter *q*<*r*>, where <*r*> is an average distance between the electron and the hole of the exciton. The absorption distribution due to dipole transitions has a maximum at *q* = 0, while the distribution of higher-order transitions showed a maximum at *q* ≠ 0. Even when the absorption intensities of multiple higher-order transitions overlap, the absorption intensity maximum should be *q* ≠ 0, which is consistent with the result of this study. The electric structure of am-SiO_2_ consists of a 2p orbital of the O atom as the top of the valence states and a 3p orbital of Si atom as the bottom of the conduction states [38]. Although electron excitation from p-orbital to p-orbital are not allowed in dipole transitions, quadrupole and higher-order transitions should be possible so that the bump intensity might be observed.

## Conclusion

A method for removing intensities originating from amorphous structures, which overlap in the AR-EELS spectra, was proposed to derive an accurate *q*-dependent dielectric function *ε*(***q***, *ω*) for amorphous materials. The overlapping intensities are the elastic scattering intensity originating from the amorphous structure *I*_amo_ela_(***q***, 0) and the inelastic scattering intensity associated with the elastic scattering *I*_amo_ela+inela_(***q***, E). These intensities were removed assuming that *I*_amo_ela+inela_(***q***, *E*)/*I*_amo_ela_(***q***, 0) corresponds to the ratio of the inelastic scattering intensity *I*_inela_(0, E) to the transmitted beam intensity *I*_trans_ in the EELS spectrum at *q* = 0 Å^−1^. From the exciton absorption intensity distribution in *q*-space estimated from the derived *ε*(***q***, *ω*), the exciton size was evaluated. By applying the proposed method, the exciton absorption intensity in the *q*-region, where the effect of the amorphous halo is dominant, was greatly suppressed, meaning derivation of the accurate exciton absorption intensity distribution in *q*-space. In addition, the exciton size was evaluated to be approximately 2 nm (±1 nm), consistent with the theoretically predicted size of ∼1 nm. These results suggest that the proposed method was effective in deriving accurate *ε*(***q***, *ω*) and evaluating the exciton size of amorphous materials using AR-EELS. The proposed method will enable the evaluation of exciton size of various amorphous materials and is expected to be useful for elucidating the mechanism of optical functionality unique to amorphous materials.

## Supplementary Material

dfae056_Supp

## Data Availability

The data that support the findings of this study are available from the corresponding author upon reasonable request.
