# Supplementary material for: Derivation method of the dielectric function of amorphous materials using angle-resolved electron energy loss spectroscopy for exciton size evaluation
Source: Microscopy (Oxf). 2025 Feb 4;74(2):117–23. doi: 10.1093/jmicro/dfae056 (PMC11957257; doi:10.1093/jmicro/dfae056)
Supplement: dfae056_Supp [file dfae056_supp.zip › suppl_data/Supplementary Data MICRO-2024-00051.docx]

**Supplementary Data MICRO-2024-00051**

1. **Measurement of specimen thickness**

Specimen thickness evaluated by applying the log-ratio method at positions 1-5 in the bellow TEM image are shown in Table 1.

**Table 1 : specimen thickness**

| Position | Thickness(nm) |
| --- | --- |
| 1 | 40 |
| 2 | 40 |
| 3 | 60 |
| 4 | 50 |
| 5 | 50 |


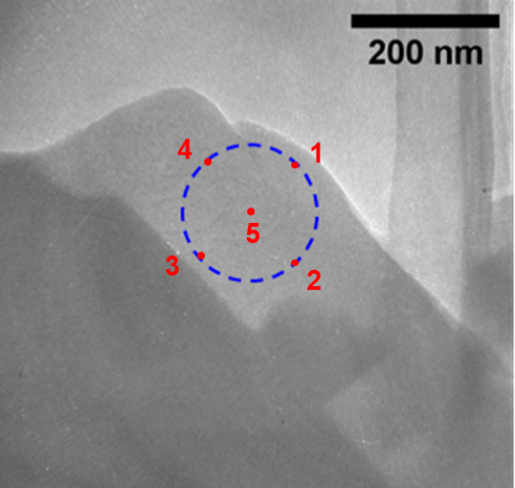


**Figure 1 : TEM image. Red dots show the positions of thickness measured.**

1. **Calculation of MFPs**

$\lambda_{inela}$ can be expressed by the following approximation for the present experimental conditions, acceleration voltage$E_{0}$= 100 kV and collection semi angle $\beta=7$ mrad. [Egerton R F (2011) *Electron Energy-Loss Spectroscopy in the Electron Microscope*, 3rd ed. (Springer).]

$$\lambda_{inela}\approx\frac{106F\left( E_{0}/E_{m} \right)}{ln\left( 2\beta E_{0}/E_{m} \right)}$$

$F$ is a relativistic factor that depends on $E_{0}$, and $E_{m}$ is a term that depends on effective atomic number $Z_{\mathrm{eff}}$.

$$F=\frac{\left( 1+\frac{E_{0}}{1022}\mathrm{keV} \right)}{\left( 1+\frac{E_{0}}{511}\mathrm{keV} \right)^{2}}=0.768$$

$$E_{m}\approx7.6{Z_{\mathrm{eff}}^{0.36}\approx=17.56, Z}_{\mathrm{eff}}\approx\frac{\sum_{i} f_{i}Z_{i}^{1.3}}{\sum_{i} f_{i}Z_{i}^{0.3}}=10.23$$

$$(Z_{i}=14\left( \mathrm{Si} \right), 8\left( O \right). f_{i}:atomic fraction)$$

From the above, $\lambda_{inela}=105$ nm was calculated.

$\lambda_{ela}$ can be expressed as$\left( \varrho N\sigma_{ela} \right)^{-1}$. [Reimer L (1989) *Transmission Electron Microscopy*, 2nd ed. (Springer).]$\varrho$ is the density of the material. $N$ is the number of atoms per unit mass. $\sigma_{\mathrm{ela}}$ is the scattering cross section for elastic scattering.

$$\sigma_{\mathrm{ela}}=\frac{1.5\times{10}^{-6}}{\beta^{2}}Z^{\frac{3}{2}}\left( 1-0.23\frac{Z}{137\beta} \right)\mathrm{for}\frac{Z}{137\beta}<1.2$$

Where $\beta=\frac{\upsilon}{c}$ and $\beta=0.55$ at 100 kV. Using the effective atomic number $Z_{\mathrm{eff}}=10.23$ for Z, $\frac{Z}{137\beta}=0.14<1.2$ is satisfied and the above equation is applicable. $\sigma_{\mathrm{ela}}=1.6\times{10}^{-4} [{nm}^{2}]$. Using $N=1.0\times{10}^{22} [/g]$, $=2.5\times{10}^{-21} \left[ \frac{g}{{nm}^{3}} \right], \lambda_{ela}=253 nm$ is calculated.

1. **Specimen thickness dependence of I_amo_ela+inela_/I_amo_ela_**

Assuming that the inelastic scattering intensities follow a Poisson distribution, each scattering intensity I is expressed as I=I_0_ t/λ exp(-t/λ) using the incident electron intensity I_0_, MFPs λ = 105 nm, and specimen thickness t = 50 nm. In this study, $\frac{\text{I}_{\text{amo\_}\text{ela+}\text{inela}}\left( \text{q}\text{, E} \right)}{\text{I}_{\text{amo\_ela}}\left( \text{q}\text{, 0} \right)}$ = $\frac{\text{I}_{\text{inela}}\left( \text{0, E} \right)}{\text{I}_{\text{trans}}}$ is assumed for I_amo_ removal, the right side is ~0.48.

The same assumption for the electrons scattered at q ≠ 0 is expressed as $\text{I}_{\text{amo\_ela}}\left( \text{q}\text{, 0} \right)$= I_0_ t/λ_ela_ exp(-t/λ_ela_) and $\text{I}_{\text{amo\_}\text{ela+}\text{inela}}\left( \text{q}\text{, E} \right)$ = I_0_ t’/λ_inela_ exp(-t’/λ_inela_). t' is the specimen thickness experienced by the $\text{I}_{\text{amo\_}\text{ela+}\text{inela}}\left( \text{q}\text{, E} \right)$ electrons as inelastically scattered electrons.


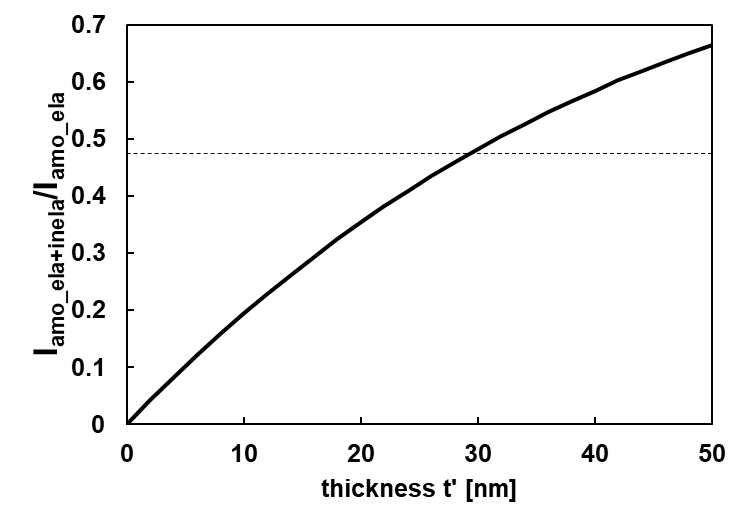


Figure 2: t’ dependence of I_amo_ela+inela_/I_amo_ela_.

The solid line in the figure shows the t' dependence of I_amo_ela+inela_/I_amo_ela_, and the dotted line shows I_inela_/I_trans_. The intersection is at t'~30 nm.
